# Improving chemical reaction yield prediction using pre-trained graph neural networks

**DOI:** 10.1186/s13321-024-00818-z

**Published:** 2024-03-01

**Authors:** Jongmin Han, Youngchun Kwon, Youn-Suk Choi, Seokho Kang

**Affiliations:** 1 Department of Industrial Engineering, Sungkyunkwan University, 2066 Seobu-ro, Jangan-gu, Suwon, Republic of Korea; 2 Samsung Advanced Institute of Technology, Samsung Electronics Co. Ltd., 130 Samsung-ro, Yeongtong-gu, Suwon, Republic of Korea

**Keywords:** Chemical reaction yield prediction, Graph neural network, Pre-training, Deep learning

## Abstract

Graph neural networks (GNNs) have proven to be effective in the prediction of chemical reaction yields. However, their performance tends to deteriorate when they are trained using an insufficient training dataset in terms of quantity or diversity. A promising solution to alleviate this issue is to pre-train a GNN on a large-scale molecular database. In this study, we investigate the effectiveness of GNN pre-training in chemical reaction yield prediction. We present a novel GNN pre-training method for performance improvement.Given a molecular database consisting of a large number of molecules, we calculate molecular descriptors for each molecule and reduce the dimensionality of these descriptors by applying principal component analysis. We define a pre-text task by assigning a vector of principal component scores as the pseudo-label to each molecule in the database. A GNN is then pre-trained to perform the pre-text task of predicting the pseudo-label for the input molecule. For chemical reaction yield prediction, a prediction model is initialized using the pre-trained GNN and then fine-tuned with the training dataset containing chemical reactions and their yields. We demonstrate the effectiveness of the proposed method through experimental evaluation on benchmark datasets.

**Supplementary Information:**

The online version contains supplementary material available at 10.1186/s13321-024-00818-z.

## Introduction

A chemical reaction is a process in which reactants are changed into products through chemical transformations. The percentage of products obtained relative to the reactants consumed is referred to as the chemical reaction yield. The prediction of the chemical reaction yields provides clues for exploring high-yield chemical reactions without the need for conducting direct experiments. This is crucial for accelerating synthesis planning in organic chemistry by significantly reducing time and cost. Machine learning has been actively utilized for the fast and accurate prediction of chemical reaction yields in a data-driven manner [1–8].

Recently, deep learning has shown remarkable performance in predicting chemical reaction yields by effectively modeling the intricate relationships between chemical reactions and their yields using neural networks. Schwaller et al. [6, 7] represented a chemical reaction as a series of simplified molecular-input line-entry system (SMILES) strings and built a bidirectional encoder representations from transformers (BERT) as the prediction model. Kwon et al. [8] represented a chemical reaction as a set of molecular graphs and built a graph neural network (GNN) that operates directly on the molecular graphs as the prediction model. The use of GNNs led to a significant improvement in the predictive performance owing to their high expressive power on molecular graphs [9, 10].

Despite its effectiveness, the predictive performance of a GNN can suffer when it is trained on an insufficient training dataset in terms of quantity or diversity. For example, a GNN may not generalize well to query reactions involving substances that are not considered in the training dataset. Although the performance can be significantly improved by securing a large-scale training dataset, this is difficult in practice because of the high cost associated with conducting direct experiments to acquire the yields for a large number of chemical reactions.

To alleviate this issue, a promising solution is to pre-train a GNN on a large-scale molecular database and use it to adapt to chemical reaction yield prediction. Various pre-training methods have been studied in the literature, which can be categorized into contrastive learning and pre-text task approaches [11, 12]. The contrastive learning approach pre-trains a GNN by learning molecular representations such that different views of the same molecule are mapped close together, and views of different molecules are mapped far apart [13–18]. Most existing methods based on this approach have utilized data augmentation techniques to generate different views of each molecule. Data augmentation may potentially alter the properties of the molecules being represented [19, 20]. The pre-text task approach acquires the pseudo-labels of molecules and pre-trains a GNN to predict them [21–25]. Existing methods have attempted to define appropriate pre-text tasks in various ways to effectively learn molecular representations. The process of acquiring pseudo-labels can be costly and time-consuming depending on how the pre-text task is defined. Since both approaches have their own advantages and drawbacks, it is important to choose the most suitable pre-training method that best aligns with the objective of a specific downstream task that needs to be addressed.

In this study, we propose a novel pre-training method, **MolDescPred**, to improve the performance in predicting chemical reaction yields. **MolDescPred** is based on the pre-text task approach to pre-train a GNN. Given a molecular database containing a substantial number of molecules, we calculate the molecular descriptors for the molecules and reduce their dimensionality by applying principal component analysis (PCA). Each molecule is then pseudo-labeled with a vector of its principal component scores. The GNN is then pre-trained to predict the pseudo-label of its input molecule. For chemical reaction yield prediction, a prediction model is initialized using the pre-trained GNN and then is fine-tuned with a training dataset composed of chemical reactions and their corresponding yields. Through experiments on benchmark datasets, we demonstrate the effectiveness of the proposed method compared to existing methods, especially when the training dataset is insufficient.

## Method

### Problem definition

For chemical reaction yield prediction, we aim to build an accurate prediction model *f* which takes a chemical reaction $(\mathcal {R}, \mathcal {P})$ as the input to predict the yield *y* by learning from the training dataset $\mathcal {D}=\{(\mathcal {R}_i, \mathcal {P}_i, y_i)\}_{i=1}^N$. Given a query chemical reaction $(\mathcal {R}_*, \mathcal {P}_*)$, the prediction model *f* can be used to make a prediction for the yield $y_*$ as:

1 $$\begin{aligned} \hat{y}_*=f(\mathcal {R}_*, \mathcal {P}_*). \end{aligned}$$

It should be noted that additional information, such as the operating conditions for chemical reactions, can be utilized as extra input for the model *f*. If we denote this additional information by $\mathcal {Z}$, the problem can be formulated as learning the model *f* from the dataset $\mathcal {D}'=\{(\mathcal {R}_i, \mathcal {P}_i,\mathcal {Z}_i, y_i)\}_{i=1}^N$. The input and output of the model *f* can be described as:

2 $$\begin{aligned} \hat{y}_*=f(\mathcal {R}_*, \mathcal {P}_*, \mathcal {Z}_*). \end{aligned}$$

The data representation used for the prediction model *f* is as follows. In a chemical reaction $(\mathcal {R}, \mathcal {P})$, $\mathcal {R}$ and $\mathcal {P}$ denote the sets of reactants and products, respectively. The set $\mathcal {R}=\{\mathcal {G}^{\mathcal {R},1},\ldots ,\mathcal {G}^{\mathcal {R},m}\}$ contains *m* reactant molecules represented as molecular graphs, where *m* can vary for each reaction. The set $\mathcal {P}=\{\mathcal {G}^{\mathcal {P}}\}$ contains a single molecular graph representing a product molecule. Each molecular graph $\mathcal {G}=(\mathcal {V}, \mathcal {E})$ represents the topology of a molecule. Here, $\mathcal {V}$ and $\mathcal {E}$ are the sets of nodes and edges associated with heavy atoms and their chemical bonds within the molecule. Hydrogen atoms are implicitly handled as node features of their neighboring heavy atoms. Each node vector $\textbf{v}^j\in \mathcal {V}$ denotes the node features regarding the *j*-th heavy atom in a molecule, including the atom type, formal charge, degree, hybridization, number of adjacent hydrogens, valence, chirality, associated ring sizes, whether it accepts or donates electrons, whether it is aromatic, and whether it is in a ring. Each edge vector $\textbf{e}^{j,k}\in \mathcal {E}$ denotes the edge features regarding the chemical bond between *j*-th and *k*-th heavy atoms, including the bond type, stereochemistry, whether it is in a ring, and whether it is conjugated.

**Fig. 1:**
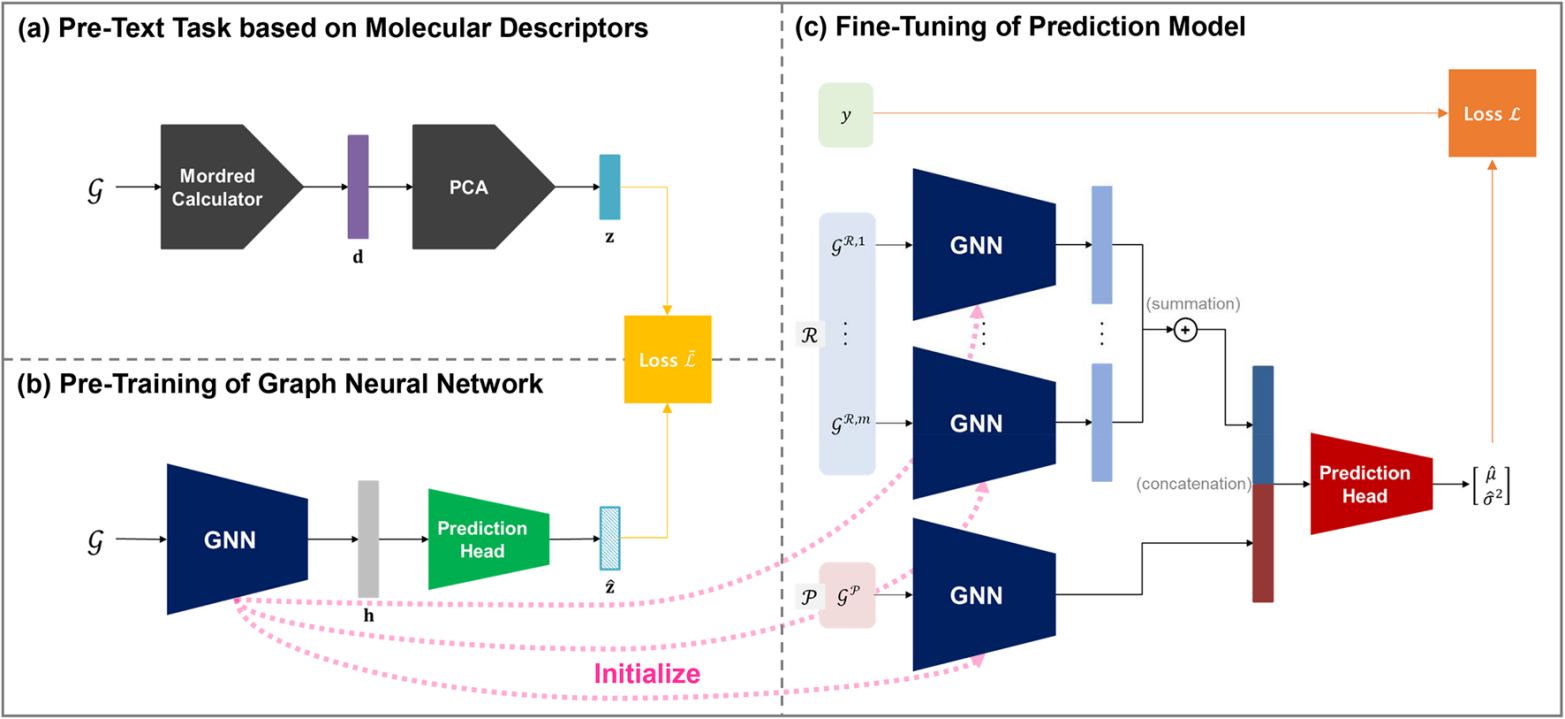
Three-phase procedure for training the prediction model with **MolDescPred**: (**a**) Molecular descriptors embedded in a reduced dimensionality are assigned as pseudo-labels to molecules in the pre-training dataset; (**b**) A GNN is pre-trained to predict the pseudo-label of each molecule in the pre-training dataset; (**c**) After initializing the GNN parameters with the pre-trained ones, the prediction model is fine-tuned using the training dataset for the target task

The objective of this study is to improve the performance of the prediction model *f*, especially in scenarios where the training dataset $\mathcal {D}$ lacks sufficient quantity or diversity. To achieve this, the proposed method **MolDescPred** employs a three-phase procedure for training the prediction model, as illustrated in Fig. 1. In the first phase, we define a pre-text task based on molecular descriptors using a large molecular database. In the second phase, we pre-train a GNN from the pre-text task. In the third phase, we incorporate the pre-trained GNN as part of the model *f* and fine-tune the model *f* using the training dataset $\mathcal {D}$. We provide a detailed description of each phase in the following subsections.

### Pre-text task based on molecular descriptors

**Fig. 2:**
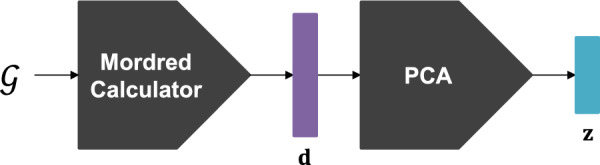
Procedure of acquiring pseudo-labels for defining a pre-text task

Molecular descriptors are numerical representations of the chemical information of a molecule derived through logical and mathematical procedures [26]. Molecular descriptors have been commonly used as inputs for prediction models in a wide range of molecular property prediction tasks [27–30]. In contrast, we utilize molecular descriptors to define a pre-text task for pre-training a GNN. Specifically, molecular descriptors embedded in a reduced dimensionality are used as pseudo-labels for the molecules. Fig. 2 illustrates the procedure of acquiring the pseudo-labels for defining a pre-text task.

Given a molecular database containing a substantial number of molecules, denoted as $\mathcal {S}=\{\mathcal {G}_i\}_{i=1}^M$, we calculate the molecular descriptors using the Mordred calculator [31]. It was originally designed to generate 1,826 molecular descriptors per molecule, including 1,613 2D and 213 3D descriptors, by leveraging a wide range of chemical and structural properties. The detailed information about the descriptors can be found in [31]. These descriptors can be efficiently calculated at high speed, with high scalability to large molecules. We exclude the 3D descriptors, assuming that molecular geometry information is not available for use in the database. For each molecular graph $\mathcal {G}$, a *p*-dimensional vector of molecular descriptors $\textbf{d} \in \mathbb {R}^p$ is obtained as:

3 $$\begin{aligned} \textbf{d} = (d_{1}, \ldots , d_{p}) = \text {Mordred}(\mathcal {G}). \end{aligned}$$

The molecular descriptor vector $\textbf{d}$ is high-dimensional and contains redundant information and noise. Thus, we apply PCA to reduce the dimensionality while preserving most of the original information [32]. The primary idea of PCA is to create new features, formed through linear combinations of the original molecular descriptors, with the objective of ensuring that these new features explain most of the variance in the molecular descriptors and are uncorrelated with each other. The objective is accomplished by eigendecomposition of the covariance matrix of the molecular descriptors calculated on $\mathcal {S}$. This yields *q* eigenvectors $\textbf{u}_1, \ldots , \textbf{u}_q$, called principal components, corresponding to the largest eigenvalues $\lambda _1, \ldots , \lambda _q$. The *j*-th eigenvalue $\lambda _j$ represents the variance explained by the *j*-th principal component $\textbf{u}_j$. To obtain a reduced *q*-dimensional vector ($q<p$), we project the original vector $\textbf{d}$ onto the *q* principal components as:

4 $$\begin{aligned} \textbf{z} = (z_{1}, \ldots , z_{q}) = (\textbf{u}_1^T\textbf{d}, \ldots , \textbf{u}_q^T\textbf{d}), \end{aligned}$$

where $z_{j}$ is the principal component score of $\textbf{d}$ obtained using the *j*-th principal component.

We establish a pre-text task by assigning each vector $\textbf{z}_i$ as a pseudo-label to the corresponding molecular graph $\mathcal {G}_i$. Subsequently, the pre-training dataset is formed as $\tilde{\mathcal {S}}=\{(\mathcal {G}_i, \textbf{z}_i)\}_{i=1}^M$.

### Pre-training of graph neural network

GNNs have shown remarkable performance in various prediction tasks in chemistry [9, 10]. GNNs are designed to operate directly on molecular graphs, enabling them to learn informative representations by effectively capturing complex relationships within molecular graphs. Among the various GNN architectures, we employ the graph isomorphism network (GIN) owing to its high expressive power when applied to molecular graphs and its widespread usage in the literature for the pre-training of GNNs [11, 33]. Specifically, we adapt a variant of the GIN proposed by Hu et al. [21] which incorporates edge features into the input representation.

The GNN processes an input molecular graph $\mathcal {G}=(\mathcal {V}, \mathcal {E})$ as follows. Each node vector $\textbf{v}^j\in \mathcal {V}$ and edge vector $\textbf{e}^{j,k}\in \mathcal {E}$ is embedded into the initial node and edge embeddings $\textbf{h}_v^{j, (0)}$ and $\textbf{h}_e^{j,k}$ using the initial node and edge embedding functions $\phi _n$ and $\phi _e$, respectively, as:

5 $$\begin{aligned}&\textbf{h}_v^{j, (0)} = \phi _n(\textbf{v}^j); \end{aligned}$$

6 $$\begin{aligned}&\textbf{h}_e^{j,k} = \phi _e(\textbf{e}^{j,k}). \end{aligned}$$

where $\phi _n$ and $\phi _e$ are parameterized as neural networks. Then, we use *L* message passing layers to iteratively update the node embeddings by aggregating information from the neighboring nodes. At the *l*-th layer ($l=1,\ldots ,L$), each node embedding $\textbf{h}_v^{j, (l)}$ is updated as:

7 $$\begin{aligned} \textbf{h}_v^{j, (l)} = \psi ^{(l)}\left( \textbf{h}_v^{j, (l-1)} + \sum _{k|\textbf{e}^{j,k}\in \mathcal {E}} \text {ReLU}(\textbf{h}_v^{j, (l-1)}+\textbf{h}_e^{j,k})\right) . \end{aligned}$$

where $\psi ^{(l)}$ is the *l*-th node embedding function parameterized as a neural network. The final node embeddings $\textbf{h}_v^{j, (L)}$ are combined via average pooling to extract a graph embedding $\textbf{h}_g$ as:

8 $$\begin{aligned} \textbf{h}_g = \frac{1}{|\mathcal {V}|}\sum _{j|\textbf{v}^j\in \mathcal {V}}\textbf{h}_v^{j,(L)}. \end{aligned}$$

Finally, the graph embedding $\textbf{h}_g$ is processed using a projection function *r* to obtain a graph-level molecular representation vector $\textbf{h}$ as:

9 $$\begin{aligned} \textbf{h} = r(\textbf{h}_g) \end{aligned}$$

**Fig. 3:**
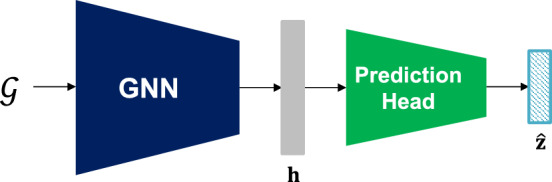
Model architecture for pre-training of GNN

In the pre-training of the GNN based on the pre-text task, we use an auxiliary prediction head to further process the graph-level molecular representation vector $\textbf{h}$ to obtain the prediction of the pseudo-label $\hat{\textbf{z}}$. It should be noted that the prediction head is used only during the pre-training phase. Fig. 3 illustrates the model architecture for the pre-training of the GNN.

Given the pre-training dataset for the pre-text task $\tilde{\mathcal {S}}=\{(\mathcal {G}_i, \textbf{z}_i)\}_{i=1}^M$, the GNN and prediction head are jointly trained using the loss function $\tilde{\mathcal {L}}$ defined as:

10 $$\begin{aligned} \tilde{\mathcal {L}}(\textbf{z}, \hat{\textbf{z}}) = \frac{1}{q}\sum _{j=1}^q \lambda _j{(z_{j}-\hat{z}_{j})}^2, \end{aligned}$$

where $\lambda _j$ denotes the eigenvalue obtained using the PCA.

### Fine-tuning of prediction model

To build the prediction model *f* for chemical reaction yield prediction, we adapt the model architecture and learning objective presented in Kwon et al.’s study [8], except that we use the GIN architecture for the GNN component in the model [34]. The model *f* takes a chemical reaction $(\mathcal {R}, \mathcal {P})$ and outputs the predictive mean $\hat{\mu }$ and variance $\hat{\sigma }^2$ for the yield *y* as:

11 $$\begin{aligned} (\hat{\mu }, \hat{\sigma }^2)=f(\mathcal {R}, \mathcal {P}). \end{aligned}$$

**Fig. 4:**
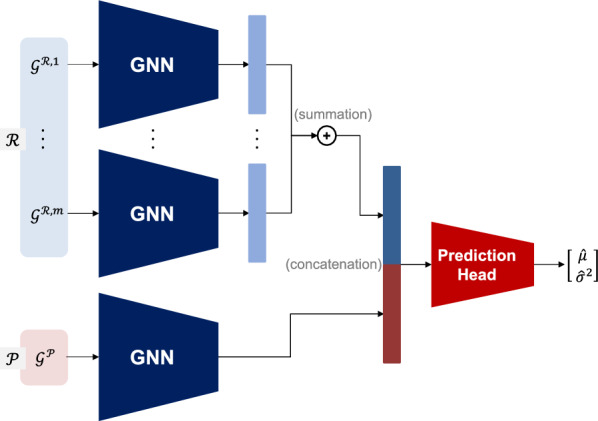
Model architecture for chemical reaction yield prediction [8]. The GNN has the GIN architecture.

The prediction model *f* consists of two main components, as illustrated in Fig. 4. First, a GNN processes each molecular graph within the input chemical reaction to obtain a molecular representation vector. Second, a prediction head integrates all molecular representation vectors to make a final prediction. To leverage prior knowledge acquired by learning the pre-text task, we initialize the GNN using the parameters obtained from the pre-training phase.

For training of the model *f*, the parameters of the GNN component are initialized using the pre-trained GNN from the previous subsection, while the remaining parameters are randomly initialized. We are provided with a training dataset for the target task $\mathcal {D}=\{(\mathcal {R}_i, \mathcal {P}_i, y_i)\}_{i=1}^N$, which comprises *N* chemical reactions and their yields. The prediction model *f* is fine-tuned using the loss function $\mathcal {L}$ as described in the referenced study [8]:

12 $$\begin{aligned} \mathcal {L}(y, \hat{\mu }, \hat{\sigma }^2)=(1-\alpha ){(y-\hat{\mu })}^2+\alpha \left[ \frac{(y-\hat{\mu })^2}{{\hat{\sigma }}^2}+\log {\hat{\sigma }}^2 \right] , \end{aligned}$$

where the first and second terms are associated with the losses under the homoscedastic and heteroscedastic assumptions, respectively, and $\alpha$ is the hyperparameter that controls the relative strength of the two terms.

### Experiments

#### Datasets

For pre-training, we used a subset of 10 million molecules extracted from the PubChem database, as provided by Chithrananda et al.’s study [35]. In the experiments, we excluded molecules that did not pass the sanity check in RDKit [36]. The molecules consisted of 25.18 heavy atoms on average, with a range of 1–891.

For chemical reaction yield prediction, we used two benchmark datasets, Buchwald-Hartwig [2] and Suzuki-Miyaura [37], which have been commonly used in previous studies to evaluate the performance of prediction models [6–8]. The Buchwald-Hartwig dataset was constructed through high-throughput experiments on the class of Pd-catalyzed Buchwald-Hartwig C-N cross-coupling reactions. It consisted of 3,955 chemical reactions and their experimentally measured yields. These reactions were generated by combining 15 aryl halides, 4 ligands, 3 bases, and 23 additives. Each chemical reaction involved 6 reactants $(m=6)$. Similarly, the Suzuki-Miyaura dataset was constructed through high-throughput experiments on the class of Suzuki-Miyaura cross-coupling reactions. The chemical reactions were generated by combinations of 15 couplings of electrophiles and nucleophiles, 12 ligands, 8 bases, and 4 solvents, resulting in a total of 5,760 chemical reactions along with their yields. The number of reactants in each chemical reaction *m* ranged from 6 to 14. The detailed operating conditions of the reactions, including temperature and pressure, were not reported in either of the benchmark datasets.

We evaluated the performance of the prediction model *f* in two different scenarios of insufficiency in the training dataset. In the quantity aspect, we utilized various training/test split ratios (70/30, 50/50, 30/70, 20/80, 10/90, 5/95, and 2.5/97.5) for both the Buchwald-Hartwig and Suzuki-Miyaura datasets. To obtain these splits, we used 10 random shuffles provided by Ahneman et al.’s study [2] for the Buchwald-Hartwig dataset and Schwaller et al.’s study [6] for the Suzuki-Miyaura dataset. In the diversity aspect, we used 4 out-of-sample training/test splits of the Buchwald-Hartwig dataset provided by Ahneman et al.’s study [2].

#### Implementation

In the phase of defining the pre-text task, we calculated 1,613 2D molecular descriptors for each molecule using the Mordred calculator [31]. The list of these 2D descriptors is provided in Additional file 1: Table S1. By eliminating descriptors with more than 10 missing values or all values being the same, 846 molecular descriptors remained $(p=846)$. All molecules with missing descriptors were excluded. Each molecular descriptor was standardized to have a mean of zero and a standard deviation of one. We then applied PCA to reduce the dimensionality of the molecular descriptors. We set the dimensionality *q* to 40, which corresponds to an explained variance of 70%. Additional file 1: Fig S1 shows the explained variance according to the reduced dimensionality determined by the number of principal components. Additional file 1: Fig S2 visualizes the principal components in relation to the original molecular descriptors, where each principal component involved a different mixture of all molecular descriptors. After dimensionality reduction, each dimension was clipped to -10 to 10 times its standard deviation and then re-standardized.

In the pre-training phase, we used a three-layer GIN architecture $(L=3)$ for the GNN. For the initial node and edge embedding functions $\phi _n$ and $\phi _e$, we used one-layer fully-connected neural networks with 300 ReLU units and 300 linear units, respectively. For the node embedding function $\psi ^{(l)}$, we used a two-layer fully-connected neural network, where each layer had 300 ReLU units. At the last message passing layer, we replaced the second layer of $\psi ^{(L)}$ with 300 linear units. For the projection function *r*, we used a one-layer fully-connected neural network with 1,024 PReLU units. For the auxiliary prediction head, we used a one-layer fully-connected neural network served as the output layer. The pre-training was performed for 10 epochs using the Adam optimizer with a batch size of 128, a learning rate of $5\cdot 10^{-4}$, and a weight decay of $10^{-5}$.

In the fine-tuning phase of the prediction model *f*, we used the pre-trained GNN obtained in the previous phase as the initialization of the GNN component in the prediction model *f*. The fine-tuning was performed using the Adam optimizer with a batch size of 128 and a weight decay of $10^{-5}$. The learning rate was initially set to $5\cdot 10^{-4}$ and decayed to $5\cdot 10^{-5}$ and $5\cdot 10^{-6}$ at the 400-th and 450-th epochs, respectively, over the entire 500 epochs.

For the inference of the prediction model *f*, we used Monte-Carlo dropout [38], following the referenced study [8]. Given a query chemical reaction, we generated 30 different predictions by conducting multiple stochastic forward passes through the model *f* with dropout activated. The final prediction for the query was obtained by averaging them.

#### Baseline methods

We conducted an exhaustive evaluation of **MolDescPred** by comparing its effectiveness with the methods presented in previous studies on chemical reaction yield prediction. For these methods, we used the configurations specified in their respective studies.

- **Multiple Fingerprint Features (MFF)** [4] represents a chemical reaction as a vector by concatenating 24 different molecular fingerprints, each generated using RDKit [36]. As a prediction model, it builds a random forest that takes this vector representation as input to predict the corresponding reaction yield.
- **YieldBERT** [6] represents a chemical reaction as a reaction SMILES string and fine-tunes a pre-trained reaction BERT model released by Schwaller et al.’s study [39] for chemical reaction yield prediction.
- **YieldBERT-DA** [7] is an improved version of **YieldBERT**, which applies data augmentation based on molecule permutations and SMILES randomization.
- **YieldMPNN** [8] represents a chemical reaction as a set of molecular graphs, similar to our study. It builds a prediction model based on a message passing neural network (MPNN) architecture [34]. Despite not utilizing any prior knowledge from pre-training, **YieldMPNN** performed better than **YieldBERT** and **YieldBERT-DA**.

For comparison of **MolDescPred** to existing pre-training methods, we evaluated different pre-training methods for initializing the GNN component in the prediction model. Compared with **MolDescPred**, the only difference was the manner in which the GNN was pre-trained. The following pre-training methods were compared. For all the existing methods, the GIN was used as the GNN architecture because they demonstrated superior performance with the GIN in the experimental results in the previous studies. The unspecified configurations for training and inference were set identical to the **MolDescPred**.

- **From-Scratch** initializes all parameters of the model *f* randomly without any pre-training. This method is similar to **YieldMPNN**, but it replaces the MPNN with GIN as the GNN architecture. The training configuration for this method is identical to that of **YieldMPNN**.
- **MolCLR** [13] pre-trains a GNN based on the contrastive learning approach. For data augmentation, it applies three graph transformation operations to generate different views of a molecular graph: atom masking, bond deletion, and sub-graph removal. The GNN learns molecular representations such that different views of the same molecular graph (i.e., positive pairs) are close and views of the different molecular graphs (i.e., negative pairs) are far apart. Because contrastive learning requires a large batch size to accommodate a large number of negative pairs, we set the batch size to 512.
- **DGI** [14] pre-trains a GNN based on the contrastive learning approach. The GNN takes a molecular graph as an input to produce node embeddings and a molecular representation vector. A discriminator is introduced to classify whether a pair of a node embedding and a molecular representation vector are associated with the same molecular graph. The GNN and discriminator are jointly trained such that the GNN learns molecular representations by maximizing the mutual information between the local node embeddings and a global molecular representation vector. Similar to **MolCLR**, we set the batch size to 512.
- **ContextPred** [21] pre-trains a GNN based on the pre-text task approach. For each node in a molecular graph, it defines a context graph as a sub-graph surrounding the neighborhood of the node. The main GNN encodes a molecular graph to obtain node embeddings that aggregates information across the neighborhood of the corresponding nodes. An auxiliary GNN, called a context GNN, is introduced to encode each context graph to obtain the context embedding. The main GNN and context GNN are jointly trained. The learning objective is the binary classification of whether a node embedding from the main GNN and a context embedding from the context GNN are associated with the same node in the molecular graph.
- **AttrMasking** [21] pre-trains a GNN based on the pre-text task approach. It randomly masks the node features in a molecular graph and assigns the masked node features as the node-level pseudo-label to the molecular graph. The GNN learns to predict the ground-truth of the masked node features in the input molecular graph.

In computational aspects, the existing methods require an auxiliary model to be maintained or involve additional repetitive operations. **MolCLR** utilizes graph transformation operations to create different views of each molecular graph and forward passes for these views at each training epoch. **DGI** requires the maintenance of the discriminator. **ContextPred** employs the auxiliary GNN. **AttrMasking** generates pseudo-labels at each training epoch. These requirements introduce extra computational costs during the pre-training phase. On the other hand, **MolDescPred** generates pseudo-labels before pre-training and trains only a single GNN with a prediction head to predict the fixed pseudo-labels during pre-training.

## Results and discussion

In the random split experiments, we conducted experiments for each training/test split ratio using 10 different random shuffles. In the out-of-sample split experiments, we repeated the experiment for each training/test split 5 times with different random seeds. We evaluated the predictive performance of each method in terms of the root mean squared error (RMSE), mean absolute error (MAE), and coefficient of determination (R^2^) calculated on the test datasets. We report the average and standard deviation of the results over repetitions. The best and second best cases are highlighted in bold and underlined font, respectively.

**Fig. 5:**
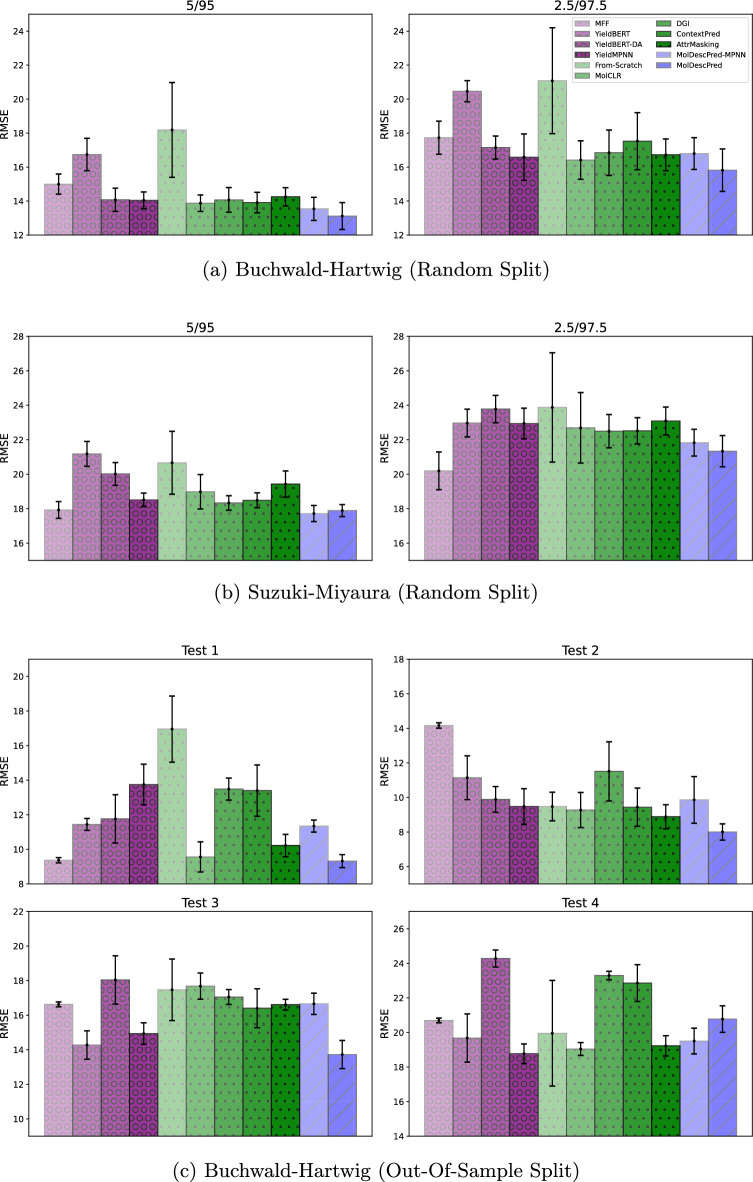
Graphical summary of RMSE comparison results: (a) Buchwald-Hartwig (Random Split), (b) Suzuki-Miyaura (Random Split), (c) Buchwald-Hartwig (Out-Of-Sample Split)

**Fig. 6:**
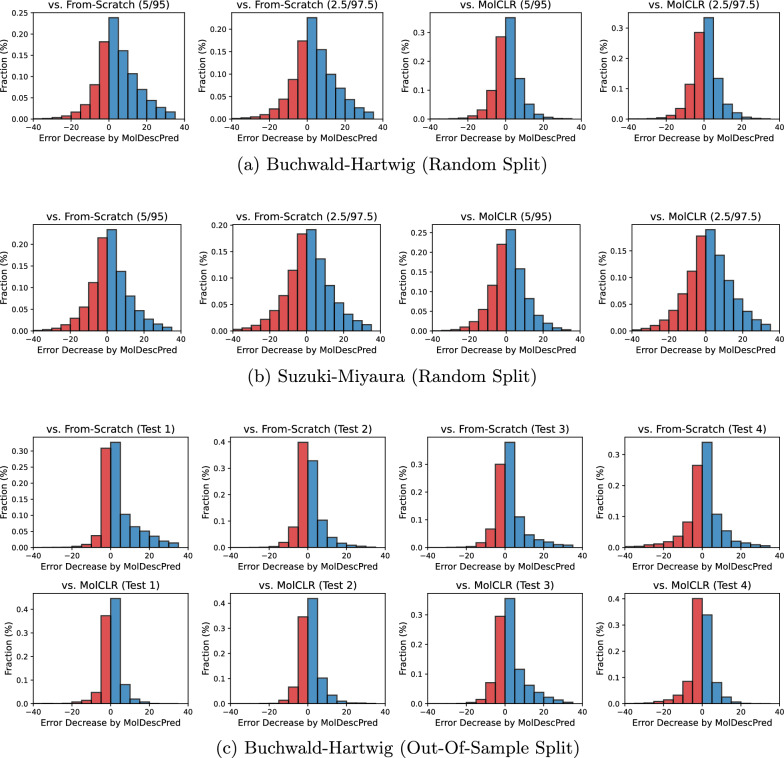
Distribution of reaction-wise error decreases by **MolDescPred**, compared to **From-Scratch** and **MolCLR**: (a) Buchwald-Hartwig (Random Split), (b) Suzuki-Miyaura (Random Split), (c) Buchwald-Hartwig (Out-Of-Sample Split)

Tables 1, 2, and 3 compare the predictive performances of the baseline and proposed methods in terms of RMSE, MAE, and R^2^, respectively. Figure 5 summarizes the RMSE comparison results using bar plots. In an overall comparison on various splits of benchmark datasets, the performance of **MolDescPred** was either superior or comparable to that of the baseline methods. For the random splits of the Buchwald-Hartwig and Suzuki-Miyaura datasets, **MolDescPred** performed the best and the second best on average, respectively. Especially, the improvement in performance was more significant when the size of the training dataset was smaller. When it comes to the out-of-sample splits of the Buchwald-Hartwig dataset, **MolDescPred** outperformed the baseline methods in 3 out of 4 splits. These results demonstrate that **MolDescPred** performed well under the insufficiency of the training dataset in terms of quantity and diversity.

All the existing GNN pre-training methods outperformed From-Scratch, indicating that the use of pre-training was helpful in improving the prediction performance. Among these methods, **MolCLR** achieved superior performance for the random splits of both the Buchwald-Hartwig and Suzuki-Miyaura datasets, but its performance slightly deteriorated on the out-of-sample splits of the Buchwald-Hartwig dataset. **AttrMasking** showed good performance in some of the out-of-sample splits. It should be noted that not all pre-training methods led to meaningful performance improvement and some of them significantly underperformed **YieldMPNN**, implying that it is important to select an appropriate pre-training method for a specific target prediction task. Figure. 6 shows the distribution of reaction-wise error decreases achieved by **MolDescPred** compared to **From-Scratch** and **MolCLR**, each of which is measured by the difference between the absolute error of **MolDescPred** and that of the compared method. The rightward skew of each distribution, characterized by a larger blue region compared to the red region, indicates that **MolDescPred** led to performance improvements in a greater number of chemical reactions within the test dataset.

Among the methods presented in the previous studies, **YieldMPNN** performed the best. **YieldMPNN** outperformed **From-Scratch**, which differs only in the GNN architecture, by a large margin in most cases. However, **YieldMPNN** performed worse than **MolDescPred**, especially on the random splits with small training datasets and out-of-sample splits. **MFF** showed low overall performance compared to the other methods, but the performance gap narrowed when using a smaller training dataset. Notably, **MFF** achieved the best performance on the 2.5/97.5 split of the Suzuki-Miyaura dataset.

To investigate the effect of the GNN architecture in the proposed method, we evaluated a variant of the proposed method, **MolDescPred-MPNN**, by using the MPNN as the GNN architecture. It can be considered as the application of the proposed pre-training to **YieldMPNN**. **MolDescPred-MPNN** yielded better performance than **YieldMPNN** in the random split experiments. While it performed significantly worse than **MolDescPred** on the Buchwald-Hartwig dataset, it surpassed **MolDescPred** on the Suzuki-Miyaura dataset. However, **MolDescPred-MPNN** performed worse than **YieldMPNN** in the out-of-sample split experiments. This indicates that the proposed method was more effective when used with the GIN.

**Table 1:** Comparison of predictive performance in terms of RMSE

Dataset	Split	Previous studies				Existing GNN pre-training methods					Proposed method	
		MFF [4]	YieldBERT [6]	YieldBERT-DA [7]	YieldMPNN [8]	From-Scratch	MolCLR [13]	DGI [18]	ContextPred [21]	AttrMasking [21]	MolDescPred-MPNN	MolDescPred
Buchwald-Hartwig (Random Split)	70/30	7.116±0.327	6.014±0.272	4.799±0.261	4.433±0.085	4.616±0.163	4.405±0.091	4.408±0.097	4.388±0.092	**4.386±0.125**	4.430±0.104	4.407±0.089
	50/50	8.051±0.322	7.288±0.198	5.877±0.348	5.387±0.202	6.088±0.982	5.279±0.167	5.364±0.222	5.327±0.183	5.328±0.216	5.326±0.231	**5.263±0.181**
	30/70	9.492±0.364	9.338±0.424	7.822±0.463	6.970±0.403	7.557±0.473	**6.837±0.387**	6.963±0.403	6.947±0.400	6.944±0.407	6.899±0.394	6.850±0.400
	20/80	10.487±0.259	10.306±0.303	9.164±0.668	8.204±0.372	9.317±0.713	**8.040±0.399**	8.271±0.498	8.175±0.333	8.268±0.398	8.093±0.365	8.043±0.426
	10/90	12.450±0.357	12.393±0.499	11.633±0.293	10.875±0.448	13.232±0.880	10.816±0.537	10.935±0.553	10.982±0.473	10.912±0.672	10.945±0.466	1**0.648±0.544**
	5/95	14.994±0.593	16.740±0.950	14.073±0.687	14.041±0.492	18.188±2.789	13.873±0.485	14.068±0.728	13.911±0.601	14.250±0.537	13.542±0.681	**13.117±0.792**
	2.5/97.5	17.731±0.970	20.463±0.623	17.151±0.677	16.586±1.364	21.081±3.116	16.414±1.134	16.845±1.334	17.526±1.680	16.722±0.938	16.798±0.935	**15.817±1.250**
	avg. rank	10.29±0.88	9.86±0.35	8.00±0.76	5.29±1.67	9.57±1.29	2.00±0.76	5.86±0.64	4.86±1.88	4.57±1.99	4.00±1.51	**1.71±1.03**
Suzuki-Miyaura (Random Split)	70/30	11.428±0.341	12.073±0.463	10.524±0.482	9.467±0.459	9.742±0.489	9.289±0.516	9.430±0.474	9.297±0.462	**9.225±0.465**	9.271±0.446	9.333±0.478
	50/50	12.208±0.169	13.148±0.270	11.797±0.250	10.225±0.135	10.691±0.171	10.155±0.142	10.222±0.191	**10.091±0.164**	10.156±0.183	10.097±0.157	10.133±0.164
	30/70	13.347±0.148	14.614±0.381	13.337±0.357	11.593±0.136	12.449±0.450	11.542±0.190	11.771±0.181	11.569±0.194	11.654±0.159	**11.507±0.175**	11.550±0.222
	20/80	14.347±0.335	15.966±0.381	14.851±0.576	12.734±0.347	14.404±0.902	12.736±0.322	13.051±0.351	12.837±0.363	12.911±0.345	**12.650±0.324**	12.717±0.225
	10/90	16.062±0.445	18.734±0.530	17.129±0.683	15.164±0.344	17.813±1.236	15.239±0.399	15.520±0.444	15.371±0.452	15.739±0.523	**14.973±0.395**	15.050±0.256
	5/95	17.927±0.484	21.181±0.724	20.016±0.661	18.511±0.392	20.665±1.823	18.982±1.000	18.332±0.421	18.487±0.431	19.430±0.760	**17.720±0.466**	17.891±0.351
	2.5/97.5	**20.199±1.096**	22.967±0.804	23.780±0.793	22.943±0.887	23.878±3.170	22.692±2.048	22.495±0.965	22.519±0.762	23.088±0.806	21.829±0.774	21.338±0.908
	avg. rank	7.14±3.40	10.57±1.05	9.29±0.45	5.43±1.68	9.14±1.12	4.29±1.58	5.71±1.16	4.14±1.36	6.00±2.39	**1.57±0.73**	2.71±1.03
Buchwald-Hartwig (Out-Of-Sample Split)	Test 1	9.369±0.151	11.441±0.342	11.761±1.398	13.746±1.175	16.956±1.913	9.559±0.871	13.484±0.636	13.398±1.480	10.219±0.646	11.343±0.346	**9.320±0.376**
	Test 2	14.163±0.155	11.144±1.267	9.886±0.741	9.476±1.027	9.474±0.829	9.274±1.016	11.511±1.711	9.439±1.103	8.883±0.697	9.860±1.349	**8.002±0.472**
	Test 3	16.629±0.141	14.276±0.820	18.041±1.395	14.939±0.622	17.471±1.777	17.681±0.757	17.053±0.429	16.404±1.127	16.608±0.310	16.659±0.616	**13.726±0.814**
	Test 4	20.698±0.135	19.679±1.397	24.279±0.494	**18.774±0.566**	19.954±3.058	19.044±0.370	23.295±0.244	22.858±1.064	19.229±0.587	19.507±0.745	20.780±0.767
	avg.rank	6.50±3.20	5.50±2.50	9.25±1.79	5.00±3.39	7.75±2.38	4.50±3.20	9.25±0.83	6.25±2.28	3.50±1.12	5.75±1.30	**2.75±3.03**

The best and second-best cases are highlighted in bold and underlined font, respectively

**Table 2:** Comparison of predictive performance in terms of MAE

Dataset	Split	Previous studies				Existing GNN pre-training methods					Proposed method	
		MFF [4]	YieldBERT [6]	YieldBERT-DA [7]	YieldMPNN [8]	From-Scratch	MolCLR [13]	DGI [18]	ContextPred [21]	AttrMasking [21]	MolDescPred-MPNN	MolDescPred
Buchwald-Hartwig (Random Split)	70/30	4.694±0.116	3.990±0.153	3.090±0.118	2.920±0.056	3.038±0.096	2.896±0.060	2.909±0.060	**2.888±0.060**	2.905±0.049	2.921±0.054	2.899±0.061
	50/50	5.370±0.134	4.792±0.124	3.744±0.150	3.497±0.090	3.957±0.796	**3.420±0.054**	3.488±0.074	3.465±0.057	3.485±0.078	3.463±0.082	3.439±0.054
	30/70	6.471±0.183	6.075±0.222	4.833±0.167	4.483±0.165	4.873±0.244	**4.400±0.152**	4.489±0.150	4.462±0.132	4.496±0.160	4.439±0.137	4.408±0.147
	20/80	7.271±0.200	6.862±0.212	5.781±0.252	5.311±0.154	6.119±0.415	5.197±0.169	5.345±0.203	5.309±0.146	5.392±0.170	5.240±0.170	**5.196±0.187**
	10/90	8.962±0.308	8.607±0.387	7.705±0.236	7.196±0.274	9.077±0.809	7.158±0.269	7.304±0.268	7.286±0.209	7.269±0.359	7.266±0.250	**7.061±0.262**
	5/95	11.085±0.322	12.117±0.789	9.651±0.338	9.677±0.408	14.043±2.879	9.932±0.408	9.688±0.467	9.614±0.393	9.716±0.392	9.434±0.418	**9.058±0.463**
	2.5/97.5	13.592±0.950	15.979±0.817	12.243±0.631	11.747±1.005	16.003±2.434	11.903±0.815	11.870±0.823	12.512±1.239	11.775±0.647	12.075±0.622	**11.304±0.952**
	avg. rank	10.29±0.88	9.86±0.35	7.43±1.50	4.71±1.58	9.71±1.16	3.00±2.39	5.71±0.88	4.29±2.05	5.43±1.50	4.00±1.69	**1.57±0.73**
Suzuki-Miyaura (Random Split)	70/30	7.904±0.169	8.128±0.344	6.598±0.270	6.116±0.223	6.323±0.245	6.038±0.264	6.096±0.263	6.053±0.253	**6.037±0.243**	6.038±0.226	6.045±0.218
	50/50	8.522±0.118	8.922±0.235	7.539±0.153	6.725±0.089	7.053±0.133	6.676±0.088	6.729±0.138	6.661±0.119	6.702±0.141	**6.629±0.112**	6.667±0.101
	30/70	9.502±0.106	10.094±0.346	8.804±0.249	7.847±0.094	8.502±0.295	7.778±0.134	7.953±0.109	7.822±0.120	7.887±0.116	**7.751±0.082**	7.793±0.147
	20/80	10.360±0.212	11.229±0.247	10.017±0.338	8.793±0.191	10.008±0.613	8.785±0.181	9.022±0.194	8.890±0.227	8.918±0.207	**8.691±0.213**	8.775±0.161
	10/90	11.890±0.268	13.528±0.395	11.954±0.443	10.739±0.211	12.839±1.154	10.863±0.249	11.017±0.304	10.948±0.320	11.171±0.330	**10.591±0.233**	10.781±0.182
	5/95	13.545±0.281	15.695±0.618	14.294±0.507	13.451±0.353	15.307±1.530	14.691±1.191	13.381±0.301	13.543±0.248	14.120±0.513	**12.934±0.364**	13.236±0.299
	2.5/97.5	**15.640±0.813**	17.666±0.496	17.587±0.690	17.189±0.813	18.289±2.538	18.129±2.291	16.928±0.737	16.817±0.467	16.997±0.716	16.324±0.593	16.114±0.697
	avg. rank	7.86±3.14	10.71±0.70	8.71±0.45	5.00±1.69	9.00±1.20	4.86±3.04	5.86±1.36	4.29±1.03	5.43±1.92	1.43±0.73	2.71±0.70
Buchwald-Hartwig (Out-Of-Sample Split)	Test 1	6.682±0.101	7.351±0.099	7.015±0.758	8.082±0.827	10.941±1.385	6.358±0.605	7.955±0.344	8.357±1.108	6.609±0.411	7.020±0.173	** 5.980±0.231**
	Test 2	9.459±0.112	7.266±0.724	6.588±0.328	6.300±0.647	6.359±0.524	6.412±0.637	7.649±0.893	6.421±0.607	5.997±0.499	6.398±0.785	**5.469±0.396**
	Test 3	10.282±0.150	9.129±0.745	11.052±0.950	8.986±0.314	11.021±1.509	11.154±0.596	10.240±0.546	9.780±1.087	10.106±0.268	10.639±0.576	**8.340±0.351**
	Test 4	14.874±0.050	13.671±1.067	18.422±0.620	**13.190±0.754**	14.414±2.982	13.231±0.266	16.719±0.598	16.084±1.174	13.910±0.320	13.616±0.597	13.870±0.393
	avg.rank	7.50±2.50	5.75±2.38	8.50±2.29	3.75±3.11	7.75±2.59	5.25±3.70	8.50±1.66	7.50±2.29	4.00±1.58	5.50±1.80	**2.00±1.73**

The best and second-best cases are highlighted in bold and underlined font, respectively

**Table 3:** Comparison of predictive performance in terms of R^2^

Dataset	Split	Previous studies				Existing GNN pre-training methods					Proposed method	
		MFF [4]	YieldBERT [6]	YieldBERT-DA [7]	YieldMPNN [8]	From-Scratch	MolCLR [13]	DGI [18]	ContextPred [21]	AttrMasking [21]	MolDescPred-MPNN	MolDescPred
Buchwald-Hartwig (Random Split)	70/30	0.932±0.008	0.951±0.005	0.969±0.004	**0.974±0.001**	0.971±0.002	**0.974±0.001**	0.974±0.001	0.974±0.001	**0.974±0.002**	**0.974±0.002**	**0.974±0.001**
	50/50	0.913±0.007	0.928±0.004	0.953±0.006	0.961±0.003	0.949±0.019	0.962±0.003	0.961±0.003	0.962±0.003	0.962±0.003	0.962±0.003	**0.963±0.003**
	30/70	0.878±0.010	0.882±0.011	0.917±0.010	0.934±0.008	0.923±0.010	**0.937±0.007**	0.934±0.008	0.935±0.008	0.935±0.008	0.936±0.008	**0.937±0.008**
	20/80	0.852±0.007	0.857±0.008	0.886±0.017	0.909±0.008	0.883±0.018	**0.913±0.009**	0.908±0.011	0.910±0.007	0.908±0.009	0.912±0.008	**0.913±0.009**
	10/90	0.791±0.011	0.793±0.016	0.818±0.009	0.841±0.013	0.763±0.032	0.842±0.016	0.839±0.017	0.837±0.014	0.839±0.020	0.838±0.014	**0.847±0.016**
	5/95	0.697±0.024	0.622±0.042	0.733±0.027	0.734±0.019	0.546±0.146	0.741±0.018	0.733±0.028	0.739±0.023	0.726±0.020	0.753±0.025	**0.768±0.029**
	2.5/97.5	0.576±0.047	0.436±0.034	0.604±0.031	0.628±0.062	0.391±0.194	0.636±0.051	0.616±0.061	0.583±0.082	0.623±0.042	0.619±0.042	**0.662±0.053**
	avg. rank	10.29±0.88	9.86±0.35	7.86±0.99	4.14±1.73	9.57±1.29	1.71±0.70	5.00±1.77	4.29±2.31	4.14±2.17	3.14±1.64	**1.00±0.00**
Suzuki-Miyaura (Random Split)	70/30	0.834±0.010	0.815±0.013	0.859±0.012	0.886±0.010	0.879±0.011	0.890±0.011	0.887±0.011	0.890±0.010	**0.892±0.010**	0.891±0.009	0.889±0.010
	50/50	0.810±0.006	0.780±0.009	0.823±0.007	0.867±0.003	0.855±0.004	0.869±0.004	0.867±0.005	**0.870±0.004**	0.869±0.004	**0.870±0.004**	0.869±0.004
	30/70	0.774±0.006	0.729±0.014	0.774±0.012	0.829±0.004	0.803±0.014	0.831±0.005	0.824±0.005	0.830±0.005	0.827±0.004	**0.832±0.005**	0.831±0.006
	20/80	0.738±0.013	0.676±0.015	0.719±0.022	0.794±0.011	0.735±0.035	0.794±0.010	0.783±0.012	0.790±0.012	0.788±0.011	**0.797±0.010**	0.794±0.007
	10/90	0.672±0.018	0.554±0.025	0.627±0.030	0.708±0.013	0.595±0.058	0.705±0.015	0.694±0.017	0.700±0.018	0.685±0.021	**0.715±0.015**	0.712±0.009
	5/95	0.592±0.022	0.430±0.040	0.491±0.034	0.565±0.018	0.454±0.103	0.542±0.048	0.573±0.020	0.566±0.021	0.520±0.038	**0.601±0.021**	0.594±0.016
	2.5/97.5	**0.481±0.057**	0.330±0.047	0.282±0.047	0.331±0.051	0.265±0.204	0.342±0.120	0.357±0.055	0.356±0.044	0.323±0.048	0.395±0.042	0.421±0.049
	avg. rank	7.00±3.30	10.57±1.05	9.29±0.45	5.14±1.81	9.14±1.12	3.86±1.81	5.71±1.16	4.00±1.41	5.71±2.60	**1.43±0.73**	2.57±1.05
Buchwald-Hartwig (Out-Of-Sample Split)	Test 1	0.882±0.004	0.824±0.010	0.811±0.047	0.744±0.042	0.609±0.086	0.876±0.023	0.755±0.023	0.756±0.051	0.859±0.018	0.827±0.011	**0.883±0.009**
	Test 2	0.727±0.006	0.829±0.037	0.866±0.020	0.876±0.026	0.877±0.021	0.882±0.026	0.816±0.056	0.877±0.030	0.892±0.017	0.866±0.038	**0.913±0.010**
	Test 3	0.650±0.006	0.741±0.030	0.585±0.067	0.717±0.024	0.610±0.081	0.603±0.034	0.631±0.019	0.658±0.049	0.650±0.013	0.648±0.026	**0.761±0.028**
	Test 4	0.388±0.008	0.444±0.077	0.157±0.034	**0.496±0.031**	0.420±0.186	0.481±0.020	0.224±0.016	0.252±0.071	0.471±0.032	0.455±0.042	0.382±0.045
	avg.rank	6.25±3.27	5.50±2.50	9.00±2.00	5.00±3.39	7.50±2.69	4.50±3.20	9.25±0.83	6.25±2.28	3.50±1.12	5.75±1.30	**2.75±3.03**

The best and second-best cases are highlighted in bold and underlined font, respectively

**Fig. 7:**
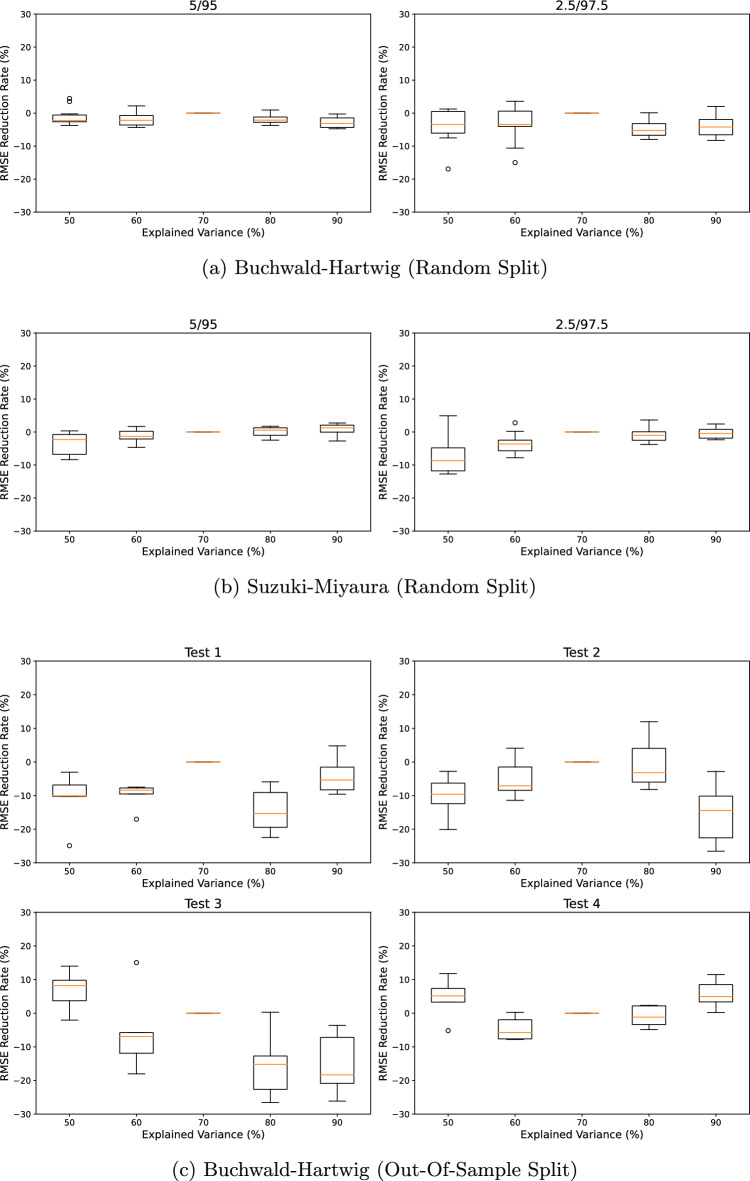
Sensitivity analysis regarding the number of principal components used in **MolDescPred**: (a) Buchwald-Hartwig (Random Split), (b) Suzuki-Miyaura (Random Split), (c) Buchwald-Hartwig (Out-Of-Sample Split)

To investigate the effect of the dimensionality of the pseudo-labels in the proposed method, we conducted a sensitivity analysis with respect to the explained variance determined by the number of principal components *q*. Figure 7 shows box plots comparing the RMSE reduction rate relative to the 70% explained variance case across various explained variances. The detailed comparison results across different levels of explained variance can be found in Additional file 1: Table S2. In the random splits of the Buchwald-Hartwig and Suzuki-Miyaura datasets, no significant differences in performance were observed. In the out-of-sample splits of the Buchwald-Hartwig dataset, while there was no clear tendency, MolDescPred demonstrated comparable performance at 70% explained variance. Therefore, it can be concluded that the current experimental setting where the dimensionality corresponds to 70% explained variance can be a reasonable choice.

## Conclusion

In this study, we presented a GNN pre-training method, **MolDescPred**, to improve the performance of chemical reaction yield prediction. The proposed method defined a pre-text task by leveraging molecular descriptors. For a molecular database, we pseudo-labeled each molecule with its molecular descriptors in a reduced dimensionality obtained through PCA. Using the database, a GNN was pre-trained to predict the pseudo-label of a molecule. The pre-trained GNN served as the initialization for the GNN component of the chemical reaction yield prediction model. By fine-tuning on the target training dataset, the prediction model achieved improved performance in predicting the yields of chemical reactions. Through experimental investigations on benchmark datasets for chemical reaction yield prediction, we demonstrated the superior performance of the proposed method over the baseline methods. The proposed method was more effective when the training dataset was insufficient in terms of quantity and diversity.

In contrast to other pre-training methods that involve repetitions of complex and expensive computations, the proposed method pre-trains a GNN to perform a simple prediction task as the pre-text task. Because the molecular descriptors can be efficiently computed on a large scale, the proposed method can be easily implemented in practical applications. One important consideration is that the molecular descriptors used to define the pre-text task are not equally beneficial for the target prediction tasks. While some descriptors may provide valuable information, others may be less useful. Guided by this intuition, a potential avenue for future work to further enhance the efficiency and effectiveness of the proposed method is to investigate ways for dynamically selecting the most advantageous molecular descriptors for specific target prediction tasks.

### Additional file

**Additional file 1:** **Table S1.** List of 2D molecular descriptors from the Mordred calculator. **Table S2.** Comparison of RMSE across various explained variances. **Figure S1.** Explained variance according to the number of principal components. **Figure S2.** Heat map visualization of principal components.

## Data Availability

We implemented the proposed method based on PyTorch in Python. The source code used in this study is available online at http://github.com/hjm9702/reaction_yield_pretrained_gnn/. The benchmark datasets are publicly accessible from https://github.com/rxn4chemistry/rxn_yields/.
