# Supplementary material for: Improving chemical reaction yield prediction using pre-trained graph neural networks
Source: J Cheminform. 2024 Mar 1;16:25. doi: 10.1186/s13321-024-00818-z (PMC10905905; doi:10.1186/s13321-024-00818-z)
Supplement: Supplementary file 1 — Additional file 1: Table S1. List of 2D molecular descriptors from the Mordred calculator. Table S2. Comparison of RMSE across various explained variances. Figure S1. Explained variance according to the number of principal components. Figure S2. Heat map visualization of principal components. [file 13321_2024_818_MOESM1_ESM.pdf]

Supplementary Information

Improving chemical reaction yield prediction using pre-trained graph neural networks

Jongmin Han[1], Youngchun Kwon[2], Youn-Suk Choi[2*], Seokho Kang[1*]

[1]Department of Industrial Engineering, Sungkyunkwan University, 2066 Seobu-ro, Jangan-gu, Suwon, Republic of Korea.

[2]Samsung Advanced Institute of Technology, Samsung Electronics Co. Ltd., 130 Samsung-ro, Yeongtong-gu, Suwon, Republic of Korea.

List of Tables

S1 List of 2D molecular descriptors from the Mordred calculator S-2
S2 Comparison of RMSE across various explained variances S-3

List of Figures

S1 Explained variance according to the number of principal components. S-3
S2 Heat map visualization of principal components. S-3

Table S1 List of 2D molecular descriptors from the Mordred calculator

Name	Number of descriptors
ABCIndex	2
AcidBase	2
AdjacencyMatrix	12
Aromatic	2
AtomCount	17
Autocorrelation	606
BCUT	24
BalabanJ	1
BaryszMatrix	104
BertzCT	1
BondCount	9
CPSA	2
CarbonTypes	11
Chi	56
Constitutional	16
DetourMatrix	14
DistanceMatrix	12
EState	316
EccentricConnectivityIndex	1
ExtendedTopochemicalAtom	45
FragmentComplexity	1
Framework	1
HydrogenBond	2
InformationContent	42
KappaShapeIndex	3
Lipinski	2
LogS	1
McGowanVolume	1
MoeType	53
MolecularDistanceEdge	19
MolecularId	12
PathCount	21
Polarizability	2
RingCount	138
RotatableBond	2
SLogP	2
TopoPSA	2
TopologicalCharge	21
TopologicalIndex	4
VdwVolumeABC	1
VertexAdjacencyInformation	1
WalkCount	21
Weight	2
WienerIndex	2
ZagrebIndex	4
Total	1,613

Table S2 Comparison of RMSE across various explained variances

Dataset	Split	Explained variance (%)				
		50	60	70	80	90
Buchwald-Hartwig (Random Split)	5/95	13.242±0.550	13.367±0.779	13.117±0.792	13.357±0.610	13.487±0.796
	2.5/97.5	16.436±1.178	16.342±1.052	15.817±1.250	16.574±1.211	16.421±1.248
Suzuki-Miyaura (Random Split)	5/95	18.501±0.616	18.080±0.441	17.891±0.351	17.871±0.417	17.736±0.386
	2.5/97.5	22.906±1.013	22.083±1.034	21.338±0.908	21.536±0.719	21.403±0.840
Buchwald-Hartwig (Out-Of-Sample Split)	Test 1	10.327±0.432	10.247±0.311	9.320±0.376	10.646±0.318	9.679±0.294
	Test 2	8.809±0.472	8.383±0.517	8.002±0.472	7.994±0.257	9.210±0.655
	Test 3	12.789±0.818	14.430±1.193	13.726±0.814	15.774±0.659	15.759±0.504
	Test 4	19.824±0.924	21.715±0.401	20.780±0.767	20.969±0.485	19.570±0.318

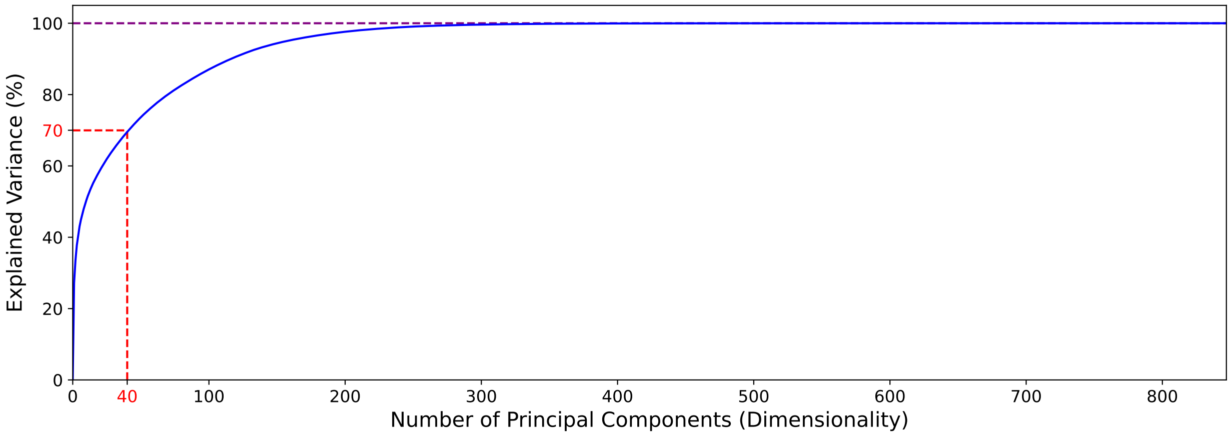

Fig. S1 Explained variance according to the number of principal components.

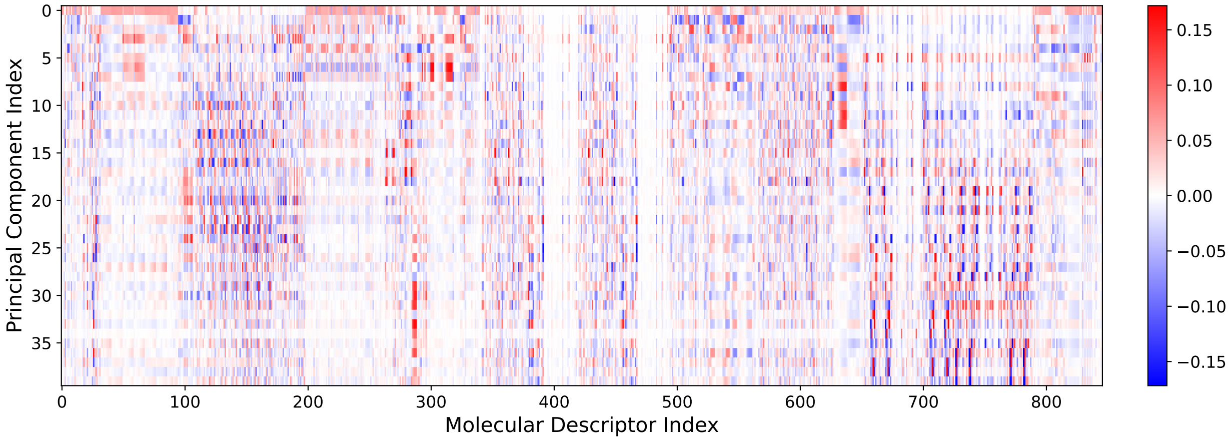

Fig. S2 Heat map visualization of principal components.
